# Chromosome-scale genomes of five Hongmu species in Leguminosae

**DOI:** 10.1038/s41597-023-02593-2

**Published:** 2023-10-17

**Authors:** Jinlong Yang, Min Liu, Sunil Kumar Sahu, Ruirui Li, Guanlong Wang, Xing Guo, Jianmei Liu, Le Cheng, Huayan Jiang, Feng Zhao, Shuguang Wei, Shixiao Luo, Huan Liu

**Affiliations:** 1 College of Forensic Science, Xi’an Jiaotong University, Xi’an, Shaanxi 710061 China; 2 State Key Laboratory of Agricultural Genomics, Key Laboratory of Genomics, Ministry of Agriculture, BGI Research, Shenzhen, 518083 China; 3 College of Life Sciences, University of Chinese Academy of Sciences, Beijing, 100049 China; 4 College of Science, South China Agricultural University, Guangzhou, 510642 China; 5 Key Laboratory of Ethnic Medical Resources Research and Southeast Asian International Cooperation in Yunnan Province, School of Tea and Coffee & School of Bioinformatics and Engineering, Pu’er University, Puer, 665000 China; 6 Key Laboratory of Ministry of Public Health for Forensic Science, Xi’an Jiaotong University, Xi’an, Shaanxi 710061 China; 7 Key Laboratory of Plant Resources Conservation and Sustainable Utilization and Guangdong Provincial Key Laboratory of Applied Botany, South China Botanical Garden, Chinese Academy of Sciences, Guangzhou, Guangdong 510650 China; 8 BGI Life Science Joint Research Center, Northeast Forestry University, Harbin, 150040 China

**Keywords:** Comparative genomics, Bioinformatics, DNA sequencing

## Abstract

The Legume family (Leguminosae or Fabaceae), is one of the largest and economically important flowering plants. Heartwood, the core of a tree trunk or branch, is a valuable and renewable resource employed for centuries in constructing sturdy and sustainable structures. Hongmu refers to a category of precious timber trees in China, encompassing 29 woody species, primarily from the legume genus. Due to the lack of genome data, detailed studies on their economic and ecological importance are limited. Therefore, this study generates chromosome-scale assemblies of five Hongmu species in Leguminosae: *Pterocarpus santalinus, Pterocarpus macrocarpus, Dalbergia cochinchinensis, Dalbergia cultrata, and Senna siamea*, using a combination of short-reads, long-read nanopore, and Hi-C data. We obtained 623.86 Mb, 634.58 Mb, 700.60 Mb, 645.98 Mb, and 437.29 Mb of pseudochromosome level assemblies with the scaffold N50 lengths of 63.1 Mb, 63.7 Mb, 70.4 Mb, 61.1 Mb and 32.2 Mb for *P. santalinus*, *P. macrocarpus*, *D. cochinchinensis*, *D. cultrata* and *S. siamea*, respectively. These genome data will serve as a valuable resource for studying crucial traits, like wood quality, disease resistance, and environmental adaptation in Hongmu.

## Background & Summary

Leguminosae (Fabaceae) is the third-largest plant family with 770 genera and 19,500 species with substantial economic value^1^. The wood of the trees is divided into the outer layers of sapwood (SW) and the inner core of heartwood (HW). HW is the inner, dark-colored wood of a tree that is a valuable commodity, particularly in the timber industry due to its high durability, exquisite color, special scent, and rot and insect resistance properties with a long history^2^. Hongmu is a special term for precious timber trees in China, comprising 29 woody species, especially the legume genus such as *Pterocarpus*, *Dalbergia*, *Senna*, and *Millettia* of the Fabaceae family^3^. Despite their high economic value and medicinal properties, the lack of genome data hampers the in-depth understanding of genetic architecture and heartwood formation mechanisms^4,5^. Therefore, in this study, we selected five highly priced and high-quality heartwood-producing trees namely *Pterocarpus santalinus*, *Pterocarpus macrocarpus*, *Dalbergia cochinchinensis*, *Dalbergia cultrata* and *Senna siamea* for generating the genomic resource.

*Pterocarpus santalinus* (2n = 20)^6^, commonly known as zitan, red sandalwood or red sanders is mainly distributed in India, South and Southwest China. The plant is valued for its heartwood with excellent red wood color, texture, decay resistance and insect resistance^7,8^. It is classified as “endangered” in the IUCN red list of threatened species, because of illegal overharvesting. Heartwood exhibits medicinal properties, including its ability to alleviate fever, reduce inflammation, combat microbes, and act as an antioxidant, all of which are harnessed in traditional medicine. The major bioactive phyto-compounds extracted from the heartwood are santalins, flavonoids, terpenoids, phenolic compounds, alkaloids, saponins, tannins, and glycosides^9^.

*Pterocarpus macrocarpus* (2n = 22)^10^, commonly known as Burma padauk, is also an important timber of Southeast Asia, with distribution in Myanmar and Thailand^11^. Its reddish HW is expensive and used for making furniture and handicrafts, because of superior wood properties, including high density and resistance to termite attack^12^.

*Dalbergia cochinchinensis* (2n = 20)^13^ (Thai Rosewood), distributed in Thailand, Cambodia, Vietnam, and Laos is listed as Critically Endangered in the IUCN red list of threatened species. Its reddish heartwood is valuable due high density, unique aroma and resistance to termites^14^.

*Dalbergia cultrata* (2n = 20) is a rosewood species also recognized as Burmese blackwood, and is distributed in a tropical and subtropical zone in Indo-China peninsula, and the south of Yunnan province in China. Heartwood is also valued for its quality, dark purplish-brown color, special scent and resistance to insects and disease. However, this species was threatened by overexploitation and listed in the IUCN red list of threatened species^15^.

*Senna siamea* (2n = 28)^16^, commonly known as kassod tree, cassod tree, and cassia tree in South or Southeast Asia, and is widely planted throughout the tropics. The HW is black-brown in texture, with high density and resistance to termites^17^. In Thailand, the young leaves and fruits are used as vegetables or traditional medicine^18^.

The heartwood properties of rot and insect resistance, durability and colors are largely defined by secondary metabolites^19^. Enhancing the quantity of secondary metabolites in the heartwood could potentially serve as a solution for reducing decay, increasing resistance to insects, and enhancing the durability of trees during breeding. Despite their economic and ecological importance, relatively little is known about the genetics and genomics of these heartwood Leguminosae species. Most previous studies have focused on molecular markers, such as microsatellites and amplified fragment length polymorphisms (AFLPs), which provide limited information about the genome structure and function^20–22^. The high-quality genome data provides a valuable resource for studying the genetic basis of important traits, such as wood quality, disease resistance, and environmental adaptation^23–27^. In this study, we provide chromosome-level genomes of five heartwood Hongmu species in Leguminosae: *Pterocarpus santalinus, Pterocarpus macrocarpus, Dalbergia cochinchinensis, Dalbergia cultrata, and Senna siamea*, using a combination of short-reads, long-read nanopore, and Hi-C data. This information can be used to improve breeding and conservation efforts for these species, as well as to develop new biotechnological applications. Additionally, the genome data can help shed light on the evolutionary history and relationships among the Leguminosae family, which is one of the largest and most diverse families of flowering plants.

## Methods

### Sample preparation and sequencing

The fresh leaves of *Pterocarpus santalinus*, *Pterocarpus macrocarpus*, *Dalbergia cochinchinensis*, *Dalbergia cultrata*, *Senna siamea* were collected form the Xishuangbanna Tropical Botanical Garden (XTBG), Yunnan, China, and were subjected for DNA extraction using CTAB (Cetyltrimethylammonium bromide) method^28^, then purified with QIAGEN Genomic kit (Cat#13343, QIAGEN). Furthermore, the DNA quality was checked by using NanoDrop (Thermo Fisher Scientific, USA) with OD260/280 ranging from 1.8-2.0 and OD260/230 between 2.0–2.2 was considered pure. Next, Qubit 4.0 (Invitrogen, USA) was used for DNA quantification. Subsequently, the long DNA fragments were selected by PippinHT system (Sage Science, USA) for each sample, and the ends were repaired by using NEBNext Ultra II End Repair/dA-tailing Kit (Cat# E7546). At last, the SQK-LSK109 kit (Oxford Nanopore Technologies, UK) was used for the adapter ligation reaction. Then the DNA libraries were performed on the Nanopore GridION X5 sequencer (Oxford Nanopore Technologies, UK). Finally, we generated 49, 44, 49, 61, and 53 Gb raw Oxford Nanopore long-reads of *P. santalinus*, *P. macrocarpus*, *D. cochinchinensis*, *D. cultrata* and *S. siamea*. The genome sequencing depth was more than 60x for each species. A total of 111, 168, 150, 155, and 162 Gb raw short insert-size reads of *P. santalinus*, *P. macrocarpus*, *D. cochinchinensis*, *D. cultrata* and *S. siamea* were generated by BGI-DIPSEQ sequencing platform. Subsequently, the extracted were digested using MboI according to the standard Hi-C library preparation protocol, then sequenced on the BGI-DIPSEQ platform, which generated 131, 132, 144, 178 and 171 Gb data for *P. santalinus*, *P. macrocarpus*, *D. cochinchinensis*, *D. cultrata* and *S. siamea*, respectively (Table S1).

For the RNAseq experiment, TIANGEN Kit was used for total RNA extraction from fresh leaves and stems. After quality control check, library construction and sequencing were performed on the Illumina platform which generated 11, 14, 22, 13, and 11 Gb raw data for *P. santalinus*, *P. macrocarpus*, *D. cochinchinensis*, *D. cultrata* and *S. siamea*, respectively, and on the other hand BGI-DIPSEQ platform generated a total of 74, 73, 69 and 77 Gb raw data for stem samples of *P. santalinus*, *D. cochinchinensis*, *D. cultrata* and *S. siamea*, respectively (Table S2).

### Estimation of genome size

The short DNA reads were used to filter the adapter, duplicated and low-quality reads by trimmomatic (v3.0)^29^ using the parameters (adapter:2:30:10:8:true LEADING:3 TRAILING:3 SLIDINGWINDOW:4:15 MINLEN:50). The clean data were used for genome size estimation based on kmerfreq 16 bit (Version 2.4) and GCE software (Table S3)^30^. The result showed that the estimated genome size ranged from 673 to 693 Mb for *P. santalinus*, 657 to 682 Mb for *P. macrocarpus*, 672 to 705 Mb for *D. cochinchinensis*, 650 to 670 Mb for *D. cultrata* and 476 to 482 Mb for *S. siamea*.

### *De novo* genome assembly and evaluation

The nanopore long reads of *P. santalinus*, *P. macrocarpus*, *D. cochinchinensis* and *S. siamea* were assembled by using NECAT^31^, while for *D. cultrata* nextdenovo^32^ software was used. Then all five assemblies were polished by short reads with NextPolish software^33^. Finally, the genomes were moved to the contig overlaps by using the purge dups (v.1.2.3)^34^ with the default parameters. As a result, we generated 623.76 Mb, 634.38 Mb, 700.50 Mb, 645.68 Mb, 437.21 Mb genome assemblies of *P. santalinus*, *P. macrocarpus*, *D. cochinchinensis*, *D. cultrata* and *S. siamea*, with the contig N50 lengths were 28.2 Mb, 12.2 Mb, 17.8 Mb, 41.1 Mb and 14.7 Mb, respectively (Table 1, Table S4).

**Table 1:** Genome assembly and assessment of five Hongmu species in Leguminosae.

Assembly		*Pterocarpus santalinus*	*Pterocarpus macrocarpus*	*Dalbergia cochinchinensis*	*Dalbergia cultrata*	*Senna siamea*
Genome-sequencing Depth (X)	Nanopore sequencing	74.21	68.21	69.75	94.48	112.81
	Short reads sequencing	165.84	255.83	213.00	239.15	341.03
	Hi-C	195.95	202.00	204.09	274.75	360.00
Estimated genome size (Mb)		673.63	657.46	705.69	650.52	476.24
Estimated heterozygosity (%)		0.58	1.13	0.97	0.85	0.45
Number of scaffolds		271	582	319	628	220
Total length of scaffolds (bp)		623,865,624	634,581,834	700,604,944	645,983,249	437,293,135
Scaffolds N50 (bp)		63,086,235	63,759,412	70,409,781	61,070,660	32,197,000
Longest scaffold (bp)		80,457,884	799,26,106	97,063,990	86,762,009	45,572,302
Number of contigs (bp)		276	486	240	42	269
Total length of contigs (bp)		623,758,624	634,382,334	700,500,444	645,687,042	437,208,635
Contigs N50 (bp)		28,158,777	12,232,783	17,819,615	41,060,504	14,728,201
Longest contig (bp)		40,322,545	39,653,416	55,196,778	81,575,240	45,588,569
GC content (%)		34.12	34.02	34.61	34.31	32.79
Mapping with Illumina reads (%)		99.18	99.29	99.83	99.34	99.65
Completeness BUSCOs (%)		97.9	98.1	97.9	97.7	97.7
Complete single-copy BUSCOs (%)		92.8	91.3	93.2	94.1	90.9
Complete duplicated BUSCOs (%)		5.1	6.8	4.7	3.6	6.8
LTR Assembly Index (LAI)		12.24	11.86	11.01	15.16	10.2

**Fig. 1:**
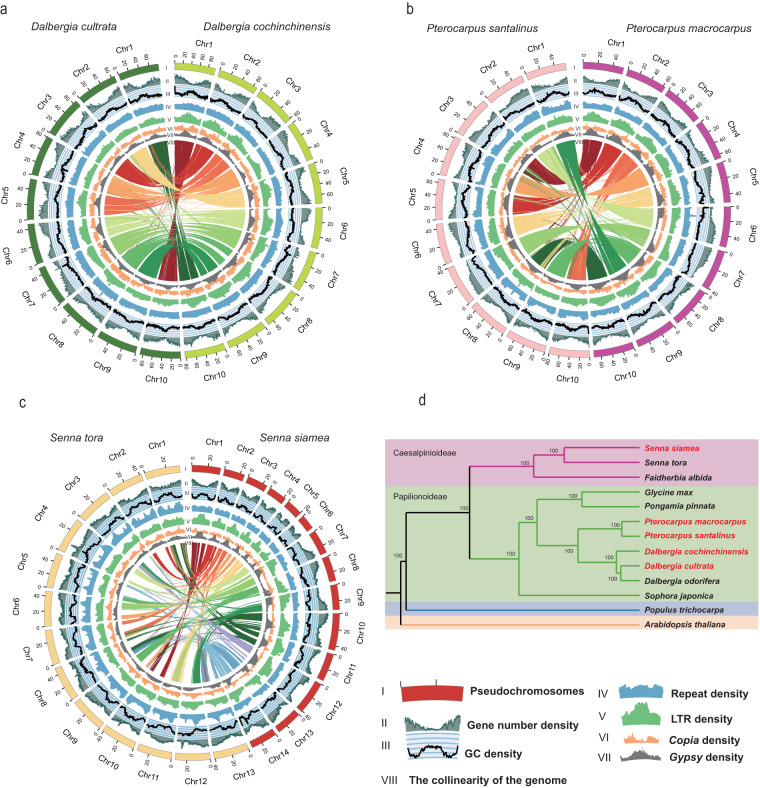
Circos plot and phylogenetic tree of five Hongmu species in Leguminosae. The distribution of genomic features along the chromosomes (scale is in Mb) (**a**–**c**). I, Pseudochromosomes. II, the density of gene number. III, the density of GC content. IV, the density of transposable elements. V, the density of transposable elements LTR. VI, the density of transposable elements LTR/Copia. VII, the density of Gypsy of LTR transposable elements. VIII, the collinearity of the genome. *Senna tora* is used for comparison only, the genome data is not generated in this study. (**d**) The phylogenetic tree of 11 representative legume species. All nodes exhibit 100% bootstrap support based on maximum likelihood analysis. All the species sequenced in the present study are highlighted in red color.

**Fig. 2:**
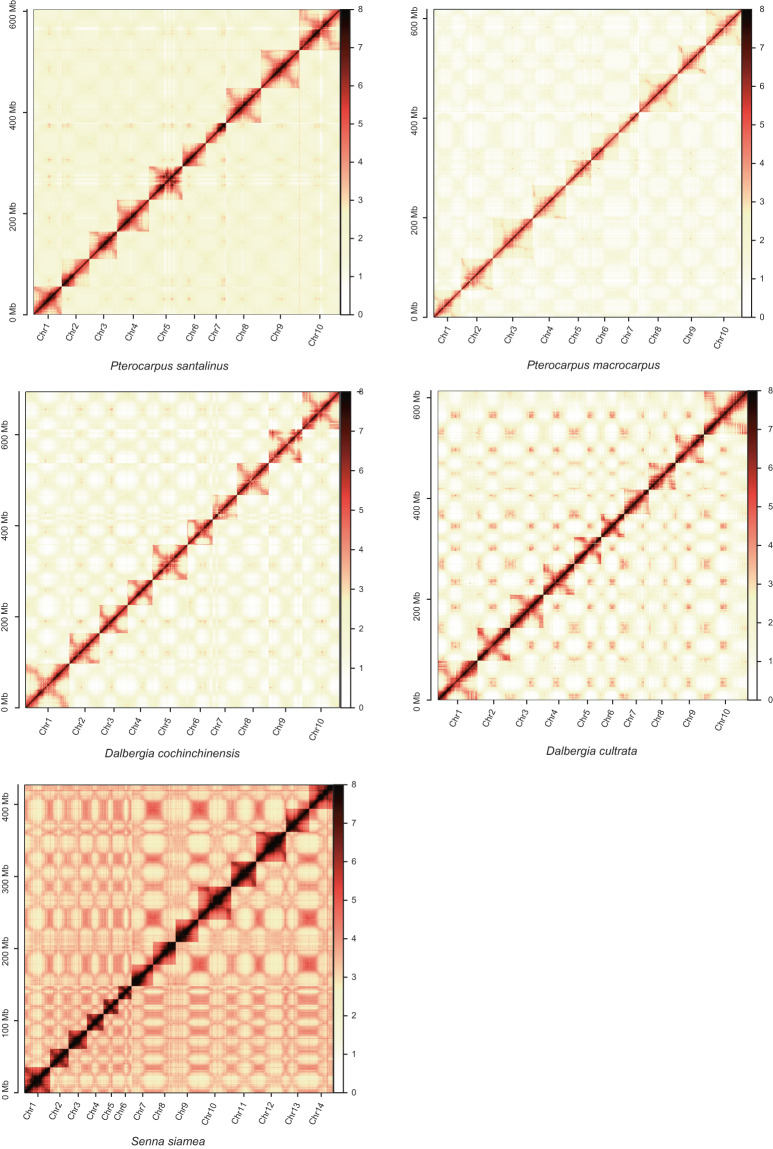
Hi-C map showing genome-wide all-by-all interactions. The map shows a high resolution of individual chromosomes that are scaffolded and assembled independently. The heat map colors ranging from white to dark red indicate the frequency of Hi-C interaction links from low to high (0–8).

Further, we used the Hi-C data to anchor the contig assemblies to the chromosomes, the Juicer software^35^ was used to extract the uniquely mapped and non-PCR duplicated Hi-C contact reads, then 3D-DNA^36^ was used to integrate the assembled genome into a pseudochromosome level assembly. Finally, the Hi-C assembly result was visualized by Juicebox and manually improved according to the Hi-C contact map. As a result, we obtained 623.86 Mb, 634.58 Mb, 700.60 Mb, 645.98 Mb, 437.29 Mb of pseudochromosome level assemblies which were anchored to 10 chromosomes in *P. santalinus*, *P. macrocarpus*, *D. cochinchinensis*, *D. cultrata*, and 14 chromosomes in *S. siamea*, with the scaffold N50 lengths of 63.1 Mb, 63.7 Mb, 70.4 Mb, 61.1 Mb and 32.2 Mb, respectively. More than 96% of scaffolds were anchored into the pseudochromosomes of each species, which is consistent with the reported chromosome number of each species (2n = 20 for *P. santalinus*, *P. macrocarpus*, *D. cochinchinensis* and *D. cultrata*, 2n = 28 for *S. siamea*) (Fig. 1a–c, Table 1, Table S4, S5, Fig. 2)

### Repeat annotation

We combined the *de novo* and homolog-based methods to find the repeat element in the genomes of five species. For *de novo* prediction, we used LTR_FINDER^37^, RepeatModeler^38^ to detect the repeat elements and then built a non-redundant library to identify the repeat element by RepeatMasker^39^. For the homolog-based methods, we used TRF to find the tandem repeats, and RepeatMasker was used to search the repeat element against the RepBase (v.21.12). In total, 49.07%, 49.49%, 62.58%, 48.88%, and 47.14% of the genome sequences were identified as repetitive sequences in *P. santalinus*, *P. macrocarpus*, *D. cochinchinensis*, *D. cultrata* and *S. siamea*, respectively (Table 2, Table S6). Long terminal repeats (LTRs) showed the highest proportions, comprising 39.49%, 39.90%, 55.88%, 43.27%, and 39.97% in *P. santalinus*, *P. macrocarpus*, *D. cochinchinensis*, *D. cultrata* and *S. siamea* respectively. The two *Dalbergia* legume wood trees showed higher LTRs than other three legume trees. Among the LTRs, the *Gypsy* LTRs (28.69%, 28.81%, 38.93%, 30.59%, 26.25%) were the most abundant in the *P. santalinus*, *P. macrocarpus*, *D. cochinchinensis*, *D. cultrata* and *S. siamea* respectively. Meanwhile, the two *Dalbergia* legume wood trees showed the highest number of *Gypsy* compared to the other three trees (Table S6).

**Table 2:** Genome annotation of five Hongmu species in Leguminosae.

Annotation	*Pterocarpus santalinus*	*Pterocarpus macrocarpus*	*Dalbergia cochinchinensis*	*Dalbergia cultrata*	*Senna siamea*
Number of predicted protein-coding genes	34,651	34,924	33,654	34,647	31,038
Average gene length (bp)	3,171.49	3,197.55	3,407.97	3,361.75	3359.51
Average exon length (bp)	226.75	223.91	225.61	227.93	214.34
Average exon number per gene	5.02	5.06	5.06	5.03	5.18
Average intron length (bp)	504.82	506.95	558.16	549.25	538.67
miRNAs	56	129	109	113	130
rRNAs	650	666	490	608	3704
tRNAs	727	828	680	551	677
Percentage of repeat sequence (%)	49.08	49.49	62.58	48.89	47.15
*Copia* (%)	6.24	6.31	13.66	9.85	6.32
*Gypsy* (%)	28.69	28.81	38.93	30.59	26.25
LINE (%)	1.65	1.86	0.78	1.09	2.91
SINE (%)	0.05	0.06	0.05	0.09	0.25
DNA transposons (%)	4.59	5.23	4.03	5.18	3.56
Percentage of Functional annotation genes (%)	98.61	96.52	99.26	99.28	92.84

### Protein-coding genes prediction and Non-coding RNA annotation

The protein-coding genes prediction was performed using BRAKER2 pipeline^40^, resulting in the identification of 34651, 34924, 33654, 34647, and 31038 protein-coding genes in *P. santalinus*, *P. macrocarpus*, *D. cochinchinensis*, *D. cultrata* and *S. siamea*, respectively (Table 2, Table S7), of which the complete BUSCOs were more than 97.7%. 97.9%, 98.1%, 97.7%, and 97.9% in these species (Table S8). All protein-coding genes were blast against NR, SwissProt, KOG, and KEGG databases with the 1e-05 E-value cutoff, resulting in 98.61%, 96.52%, 99.26%, 99.28%, and 92.84% functionally annotated genes (Table S9).

Ribosomal RNA (rRNA) genes were searched against the plant rRNA database by using BLAST. MicroRNAs (miRNA) and small nuclear RNA (snRNA) were searched against the Rfam 12.0 database. tRNAscan-SE was also used to scan for tRNAs^41^. As a result, a total of 2005, 2283, 1654, 1659, and 5437 ncRNAs were identified in *P. santalinus*, *P. macrocarpus*, *D. cochinchinensis*, *D. cultrata* and *S. siamea* genome, respectively (Table S10). In particular, the number of rRNAs in *S. siamea* was higher than the other four legume wood trees.

### Analysis of the phylogeny tree

OrthoFinder (v2.3.14)^42^ software was used for comparative genome analysis between the predicted protein-coding sequences of 13 representative plant species (including *P. santalinus*, *P. macrocarpus*, *D. cochinchinensis*, *D. cultrata*, *S. siamea*, and other published genomes of *Dalbergia odorifera*, *Glycine max*, *Pongamia pinnata*, *Sophora japonica*, *Senna tora*, *Faidherbia albida*, *Populus trichocarpa* and *Arabidopsis thaliana*) (TableS 11).

The sequences of each 302 low-copy orthogroups were extracted and aligned by MAFFT (v 7.310)^43^ after filtering the gaps. The aligned coding protein sequences of each species were then concatenated to a supergene sequence. The phylogenetic tree was subsequently constructed by IQ-Tree (v 1.6.1)^44^ with the parameters ‘-bb 1000 -alrt 1000’ (Fig. 1d).

## Data Records

All the genomic sequencing raw data are deposited in the Genome Sequence Archive in National Genomics Data Center (NGDC) Genome Sequence Archive (GSA) database with the accession number CRA011389^45^ under the BioProject accession number PRJCA017486^46^. The Chromosome-scale genome assemblies were submitted to the GenBank at NCBI under the accession number GCA_031439595.1^47^, GCA_031439585.1^48^, GCA_031216125.1^49^, GCA_031216105.1^50^, GCA_031216115.1^51^ of *P. santalinus*, *P. macrocarpus*, *D. cochinchinensis*, *D. cultrata* and *S. siamea*, respectively. The raw sequencing data are also submitted to the CNGB Sequence Archive (CNSA) of China National GeneBank DataBase (CNGBdb) under accession No. CNP0003804. Genome annotation of gene structure is available via Figshare^52^.

## Technical Validation

The completeness and contiguity of genomes were assessed by BUSCO (V3.0.2)^53^ software with the Embryophyta odb10, and the analysis suggested 97.9%, 98.1%, 97.9%, 97.7%, and 97.7% of complete embryophyte BUSCOs in the genome of *P. santalinus*, *P. macrocarpus*, *D. cochinchinensis*, *D. cultrata* and *S. siamea*, respectively (Table S12). The DNA short reads were mapped to the genomes by BWA (v.2.21) and showed a high mapping rate to the genome (99% for *P. santalinus*, *P. macrocarpus*, *D. cultrata*, *S. siamea*. 98.83% for *D. cochinchinensis*), the RNA short reads were also showed more than 90% mapping rate to the genome by using HISAT2 (V.2.1.0) (Table S13).

Furthermore, LAI (LTR Assembly Index) was used to evaluate the contiguity of the genome assembly by assessing the assembly of LTR sequences. First, LTRharvest^54^ was used to detect the LTR sequences with the parameter ‘-minlenltr 100 -maxlenltr 7000 -mintsd 4 -maxtsd 6 -motif TGCA -motifmis 1 -similar 85 -vic 10 -seed 20’, then combined with the previous LTR_FINDER result. Finally, the LTRretriever (v.2.8)^55^ was used to obtain the high-confidence LTR retrotransposons with default parameters. At last, the LAI score was calculated by using the LTRretriever with the default settings. The LAI values were 12.24, 11.86, 11.01, 15.16, and 10.2 for *P. santalinus*, *P. macrocarpus*, *D. cochinchinensis*, *D. cultrata* and *S. siamea*, respectively (Table S14). The high quality, contiguity, and completeness of the assembled genome were supported by various evidences^56^.

### Supplementary information

Description of Additional Supplementary Files

Supplementary Tables

## Data Availability

The parameters of the software were default. No specific script was used in this work.
