# Supplementary material for: Chromosome-scale genomes of five Hongmu species in Leguminosae
Source: Sci Data. 2023 Oct 17;10:710. doi: 10.1038/s41597-023-02593-2 (PMC10582184; doi:10.1038/s41597-023-02593-2)
Supplement: Supplementary file 1 — Description of Additional Supplementary Files [file 41597_2023_2593_MOESM1_ESM.docx]

**Description of Additional Supplementary Files**

Table S1. DNA sequencing statistics.

Table S2. RNA sequencing statistics.

Table S3. Genome survey.

Table S4. The genome assemly results.

Table S5. The chromosomes length of five wood species.

Table S6. The repeat element annotation.

Table S7. The result of gene annotation.

Table S8. Busco assessment of gene annotation.

Table S9. The functional annotation.

Table S10. Summary of the annotation of ncRNA in five species.

Table S11. The species used for phylogeny tree construction.

Table S12. Busco assessment of genome assembly of nanopore assembly.

Table S13. Reads mapped to the genome.

Table S14. The LAI scores.
